# Numerical simulation of magnetic drug targeting for lung cancer therapy using a bulk superconducting magnet

**DOI:** 10.1080/10717544.2025.2490836

**Published:** 2025-04-29

**Authors:** Zhenyang Xu, Tayebeh Mousavi, Mark Ainslie

**Affiliations:** Department of Engineering, King’s College London, London, UK

**Keywords:** Magnetic drug targeting, numerical simulation, capture efficiency, particle deposition efficiency, bulk superconducting magnet

## Abstract

Primary bronchus cancer is one kind of lung cancer with a very high mortality rate. Magnetic drug targeting (MDT) technology could concentrate drugs in a specific area, which could have useful application in lung cancer therapy. Due to a bulk superconducting magnet’s ability to generate a superior magnetic field strength and gradient in comparison to conventional permanent magnets, there is great potential for achieving MDT external to the body. However, current research in this area is still in its infancy, and numerical simulations exploring the guidance ability of this technology have been limited to only two-dimensional geometries, which limits further exploration toward clinical transformation. In this work, a three-dimensional lung and bulk superconducting magnet model have been built in the finite-element software package COMSOL Multiphysics. The model is used to simulate the drug delivery process in the lung via the superconducting magnet. The influence of various parameters on the capture efficiency is investigated, including lung-magnet distance, bulk superconductor properties, particle properties, and physiological or tumor structural parameters. The results demonstrate that the bulk superconducting magnet can effectively improve the capture efficiency of magnetic drugs or drug carriers within a suitable distance outside of the body, which could potentially guide the design of a practical, external superconducting MDT system in the near future.

## 1. Introduction

Lung cancer is a common cancer with a high mortality rate (Li et al. 2023; Malvezzi et al. 2023). The use of surgery, systemic chemotherapy, and systemic radiotherapy to treat this type of cancer inevitably has side effects and high risks (Garon et al. 2015; Saadat et al. 2020; Bhat et al. 2022). However, targeted therapy can concentrate drugs near diseased tissues, thereby improving treatment success and overcoming drug systemic toxicity (Wei et al. 2014; He et al. 2021; Li et al. 2024; Liu et al. 2024). Therefore, the use of targeted therapy for lung cancer has attracted the interest of many researchers (Saadat et al. 2020; Bhat et al. 2022).

Drug targeting is mainly divided into passive targeting and active targeting (Kumari et al. 2016; Sulttan and Rohani 2023). Passive targeting is mainly based on drug particle size, surface properties, and targeted tissue properties to accumulate the drug at the target location (Li et al. 2023; Sulttan and Rohani 2023). Active targeting aims to achieve directional guidance of drug particles to reach the target location by adjusting pH (Liu et al. 2014), ultrasound (Bouakaz and Escoffre 2024), magnetic field (Varmazyar et al. 2020; Qiao et al. 2023), and so on. Within the sphere of active drug delivery systems, the magnetic drug targeting (MDT) system is considered to be one of the most promising (Liu et al. 2019; Wu et al. 2023; Chen et al. 2024). Thus, the application of MDT to treat lung and other cancers is being actively pursued.

The magnetic field in MDT is mostly generated by an external magnetic source located outside the body or by a magnet implanted near the target area inside the body (Manshadi et al. 2018; Liu et al. 2019; Aryan et al. 2022). For conventional permanent magnets (the most commonly used magnet type, such as neodymium-based ones), due to the limitations of the magnetic field strength and gradient of these materials, many studies focus on implanting magnets near the target position to maximize the MDT guidance efficiency. Fernández-Pacheco et al. (2007) proposed a method of implanting permanent magnets into target organs using laparoscopic technology and conducted drug delivery experiments in New Zealand rabbits. The experiments showed that this method can effectively concentrate a sufficient amount of magnetic nanoparticles into the target area. Lindemann et al. (2021) used computer simulations to study the SPIONs guiding capabilities of Halbach magnet arrays with different sizes and combinations in multi-branch blood vessels. The results showed that the guidance is more effective within 200 µm of the target position. For breast cancer treatment, Sulttan and Rohani (2023) used a comprehensive mathematical model to predict the dasatinib magnetic nanomicelles trajectories in the internal thoracic artery under the effect of the magnet, then explored the effects of magnetic field strength, relative permeability, magnet size, drug particle size, and initial position of drug particles on guidance efficiency. A detailed summary of related research can be found in Table S1 in Supplementary Material 1.1. The magnetic field strength using conventional technology (permanent magnets or electromagnets) is quite weak, so the magnet needs to be placed very near the diseased organ, making it difficult to achieve drug targeting without compromise and/or invasive surgery, limiting widespread clinical application. It is quite complex and risky to implant permanent magnets near human organs and in deep tissues, and the process of implanting permanent magnets into the body will inevitably cause damage to the body. Therefore, it is desirable to have a suitably strong magnetic field source, such that the magnetic source could be located outside the body to achieve efficient MDT, while having almost no impact on the human body (>10 T ultra-high field MRI proves that high-intensity magnetic fields can have little impact on the human body (Moser et al. 2017; Wada et al. 2010)).

Compared with conventional permanent magnets, bulk superconducting magnets can provide a stable magnetic field on the magnet surface that is an order of magnitude higher than conventional permanent magnets. Indeed, magnetic fields have been ‘trapped’ in bulk high-temperature superconductors exceeding 17 T (Huang et al. 2020). Thus, these magnets have the potential to provide sufficient magnetic field strength and gradient to capture magnetic particles at long distances (Durrell et al. 2018). Some promising research on using bulk superconducting magnets for drug delivery has been carried out already: in particular, between 2007 and 2013, Japanese researchers conducted preliminary explorations on this topic. Takeda et al. (2007) compared the magnetic field gradients generated by NdFeB magnets and bulk superconducting magnets, and proposed that in a pig blood flow model, bulk superconducting magnets can accumulate particles at a distance of at least 20 mm. Yoshida et al. (2007) demonstrated experimentally that under the interaction of a bulk superconducting magnet with a maximum surface magnetic flux density of 2 T, the aggregation of magnetic particles over distances of ≤45 mm and ≤20 mm for distilled water and pig blood, respectively. Mishima et al. (2007) tested the guidance efficiency of two bulk superconducting magnet combinations for magnetic particles in Y-shaped blood vessels in static, translational, and rotational motions. The results showed that the guidance efficiency was the highest in the case of rotational motion. Terada et al. (2008) observed that using NdFeB (with 0.3 T surface magnetic flux density) on the surface of a capillary model can effectively achieve magnetic particle aggregation through magnetic drug accumulation simulations. Furthermore, they found that a bulk superconducting magnet (with a maximum surface magnetic flux density of 2.5 T) placed 30 mm away from the capillary surface can achieve a similar guidance effect based on the calculation of magnetic field intensity. Nakagawa et al. (2012) developed a two-dimensional linear blood vessel model and calculated the motion trajectory and accumulation efficiency of 10 magnetite liposomes (10–25 mm away from the magnet surface) under the effect of magnetic force generated by a bulk superconducting magnet (with a maximum surface magnetic flux density of 4.5 T). They observed experimentally that the drug concentration in a specific area was three times that of the control group without magnets. However, these studies mainly focus on qualitative or simple quantitative descriptions of the ability of bulk superconducting magnets to achieve MDT experimentally, such as glass blood vessel models, and so on; there are almost no quantitative studies on the MDT guidance ability of bulk superconducting magnets for specific organs or tissues. At the same time, computer-based MDT simulations have mainly focused on simple two-dimensional models, which are quite different from reality in animals, therefore, making it difficult to effectively guide the design of magnetic nanoparticle guidance equipment exploiting bulk superconducting magnet technology.

To address this challenge, three-dimensional lung and bulk superconducting magnet models have been built in the finite-element software package COMSOL Multiphysics to simulate the MDT process via a bulk superconducting magnet for lung cancer therapy. The influence of various parameters on the capture efficiency is investigated, including lung-magnet distance, bulk superconductor properties, particle parameters, and physiological or tumor structural parameters, which provides a preliminary exploration of the potential of bulk superconducting magnets in MDT applications to guide the design of practical systems exploiting this remarkable technology.

## 2. Materials and methods

### 2.1. Theoretical background

Simulating the bulk superconducting magnet as the magnetic field source, its electromagnetic properties satisfy Ampere’s law (1), and current conservation (2).

(1) $$\begin{array}{c} \nabla \times H=J \end{array}$$

(2) $$\begin{array}{c} \nabla \cdot J=0 \end{array}$$

where **H** is the magnetic field strength and **J** is the current density.

The bulk superconducting magnet is considered to be fully magnetized (without flux creep) during the drug delivery process, and the so-called trapped magnetic field can be represented by the following equation based on Bean’s critical state model and Biot–Savart law (Bean 1962; Ainslie et al. 2018; Ainslie and Fujishiro 2019):

(3) $$\begin{array}{c} B_{T}=k\mu_{0}J_{c}a \end{array}$$

where *B*_T_ is the peak trapped magnetic flux density at the center of the top surface of a *c*-axis oriented, single-grain bulk high-temperature superconductor; *µ*_0_ is the magnetic permeability of free space; *J*_c_ is the superconducting material’s critical current density; *a* is the radius of the superconducting magnet, and *k* is the simple Bean approximation correction factor, calculated as follows based on sample thickness *t* and *a*.

(4) $$\begin{array}{c} k=\frac{t}{2a}\text{ln}\;\left(\frac{a}{t}+\sqrt{1+{\left(\frac{a}{t}\right)}^{2}}\right) \end{array}$$

The magnetic field around the magnet can be calculated from Equation (5) :

(5) $$\begin{array}{c} \nabla \cdot (\mu_{0}\mu_{r}(H+M))=0 \end{array}$$

where *µ_r_* is the relative magnetic permeability and **M** is the magnetization. For human tissue and air, **M** is 0. Therefore, Equation (5) can be written as:

(6) $$\begin{array}{c} B=\mu_{0}\mu_{r}H \end{array}$$

Assuming the human body is resting, the airflow in the human lung is laminar, so the continuity and momentum Equations (7) and (8) are used (Kamali et al. 2016).

(7) $$\begin{array}{c} \rho \nabla \cdot (u)=0 \end{array}$$

(8) $$\begin{array}{c} \rho_{a}(u\cdot \nabla )u=-\nabla p+\eta \nabla \cdot (\nabla u)+F \end{array}$$

where *ρ*_a_ is the density of the air and **u** is the velocity vector of the air flow. *p* is the air pressure, and **F** is the sum of the external volumetric forces.

For the drug particles in the lung branch, their motion can be expressed by Newton’s second law:

(9) $$\begin{array}{c} \frac{d(m_{p}v)}{\text{dt}}=\sum F_{t} \end{array}$$

(10) $$\begin{array}{c} m_{p}=\frac{1}{6}\pi d_{p}^{3}\rho_{p} \end{array}$$

Here *m*_p_ is the mass of the particle, which can be calculated from the density *ρ*_p_ and diameter *d*_p_ of the particle through Equation (10). **v** is the particle velocity, and ∑**F**_t_ refers to the sum of the forces acting on the particles. For the moving particles in this work, the acting forces include drag, magnetophoretic, lift, unsteady flow, Brownian and gravitation forces. However, except for the drag and magnetophoretic forces, the other forces are considered small enough to be ignored. As a result, in our model, ∑**F**_t_ is calculated as the sum of the drag force **F**_d_ and the magnetophoretic force **F**_m_.

Then the following equations, based on the Schiller-Naumann model, can be used to calculate **F**_d_ (Loth 2008):

(11) $$\begin{array}{c} F_{d}=\frac{3\eta C_{D}{\text{Re}}_{p}}{4\rho_{p}d_{p}^{2}}m_{p}(u-v) \end{array}$$

(12) $$\begin{array}{c} C_{D}=\frac{24}{{\text{Re}}_{p}}(1+0.15{\text{Re}}_{p}^{0.687}) \end{array}$$

(13) $$\begin{array}{c} {\text{Re}}_{p}=\frac{\rho |u-v|d_{p}}{\eta } \end{array}$$

where *C*_D_ is the drag coefficient and Re_p_ is the Reynolds number of the particles.

**F**_m_ can be calculated by Equation (14) (Gao et al. 2007):

(14) $$\begin{array}{c} F_{m}=\frac{1}{4}\pi d_{p}^{3}\mu_{0}\mu_{r}(\frac{\mu_{r,p}-\mu_{r}}{\mu_{r,p}+2\mu_{r}})\nabla H^{2} \end{array}$$

where *µ*_r,p_ is the relative magnetic permeability of the particles.

### 2.2. Model definition

**Figure 1.**
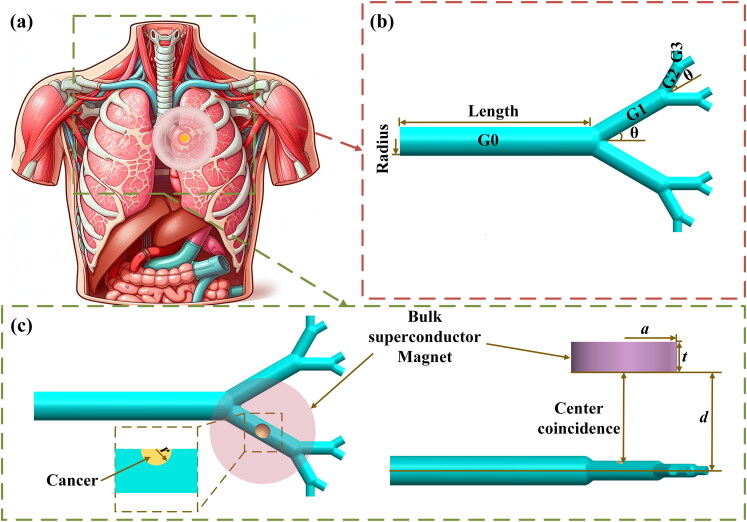
Schematic diagram of (a) the MDT process using a bulk superconducting magnet, (b) Weibel model, and (c) bulk superconductor magnet and modified Weibel model.

Figure 1(a) shows a basic schematic of the MDT process using a bulk superconducting magnet. In this study, a bulk superconducting magnet system is assumed to be placed on the surface of the skin outside the body, corresponding to the cancer area. In such a system, the bulk superconducting magnet is placed within a suitable sample holder (Tsui et al. 2022) and cryogenically cooled within a vacuum space, preventing contact between the low-temperature bulk superconducting magnet and the skin. As shown in Figure 1(b), the generations G0–G3 in the Weibel model (Weibel et al. 1963) were used to simulate the lung structure. The dimensions of each part of the model are given in Table 1. In order to simulate the distribution of cancer tissue (such as squamous cell cancer) that starts growing from the epithelium and gradually blocks the airway in the lung, the model was modified into the three-dimensional lung model shown in Figure 1(c), in which the yellow-coloured part is the cancer, and the ration between the tumor radius (*r*) and the radius of the G1 branch (*R*_G1_) *r*/*R*_G1_ = 0.8 (Manshadi et al. 2019; Sabz et al. 2019).

**Table 1.** Weibel model dimensions.

Branch	Length (mm)	Radius (*R*_GX,_ mm)
G0	120	9
G1	47.6	6.1
G2	19	4.15
G3	7.6	2.8

In this work, we assume a fully-magnetized, large single-grain Gd–Ba–Cu–O bulk superconducting magnet as the magnetic field source. The distance (*d*) between the surface of the bulk superconductor and the center plane of the lung model was set from 40 to 130 mm in order to explore the possibility of superconducting magnets to guide magnetic particles external to the body (see Supplementary Material 1.2).

During the simulation process, a three-dimensional CAD model of the superconducting magnet and lung was imported into COMSOL Multiphysics. COMSOL’s AC/DC module was used to calculate the magnetic field, where the Magnetic Fields (mf) interface was used to calculate the magnetic field generated by the bulk superconducting magnet, and the Magnetic Fields, No Current (mfnc) interface was used to calculate the magnetic field around the magnet. The CFD module was used to calculate the flow process of gas in the bronchus. The Particle Tracing module was used to calculate the trajectory of particles under composite fields. 10,000 particles were introduced into the model for the simulations. Table 2 details the material parameters and the boundary conditions. In the simulation process, the stationary solver is first used to solve for the flow field and magnetic field, then a time-dependent solver is used to solve the particle motion under the magnetic field. After each simulation, the particle deposition efficiency (PDE) of the particles at the target point is calculated by Equation (15) (Manshadi et al. 2019).

(15) $$\begin{array}{c} \text{PDE = }\frac{\text{Number of particles at the target position}}{\text{Total number of particles entering the branch}}\times 100\text{\%} \end{array}$$

**Table 2.** Magnetic particles parameters and boundary conditions.

Property	Air	Particle
Density (kg/m^3^)	1.22(Pourmehran et al. 2016)	5230(Manshadi et al. 2018)
Dynamic viscosity (Pa·s)	1.78 × 10^−5^(Pourmehran et al. 2016)	N/A
Relative magnetic permeability	1	9(Sulttan and Rohani 2023)
Particle diameter (µm)	N/A	4(Manshadi et al. 2019)
Inlet velocity (L/min)	15(Zhang et al. 2005)	Based on air velocity
Wall	No-slip	Stick
Outlet	Pressure outlet	Freeze

### 2.3. Model validation

To determine the effectiveness of this numerical model, the model of the magnet guiding particle motion in the lung and the model of the superconducting magnet were verified, respectively.

**Figure 2.**
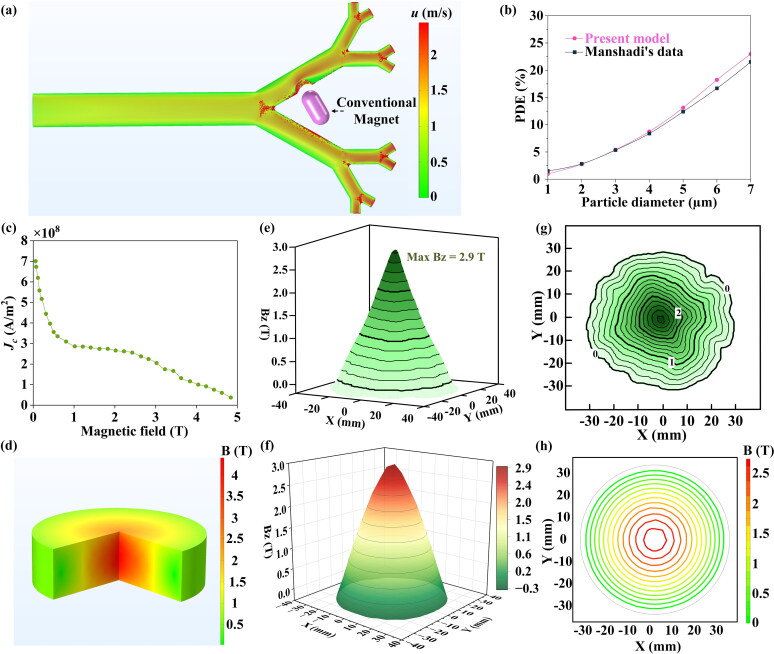
(a) Schematic diagram of validation model simulation results (particle diameter = 6 μm). (b) comparing our simulation results with Manshadi’s data (Manshadi et al. 2019). (c) *J*_c_(*B*) curves at 77 K for Gd-Ba-Cu-O bulk. (d) Simulated magnetic field distribution of the bulk superconducting magnet. Trapped field distribution at 1.2 mm above the surface of the Gd-Ba-Cu-O bulk superconducting magnet at 77 K. Three-dimensional plot of (e) experimental result, and (f) simulation result. contour plot of (g) experimental result, and (h) simulation result. (c), (e), (g) reproduced from (Nariki et al. 2005).

For the validation of the MDT model, we inputted the capsule-shaped conventional magnet with 1 T magnetic field strength in the same position as Manshadi et al. (2019) (Figure 2(a)). In addition, the material parameters were kept the same as Manshadi et al. (2019). As shown in Figure 2(b), we compare our PDE results with their data, which have good agreement.

Based on the experiments reported by Nariki et al. (2005), the bulk superconducting magnet model is assumed to be 32.5 mm in radius and 19 mm in thickness. The operating temperature is assumed to be 77 K and the magnetic field dependence of the superconductor’s critical current density, *J*_c_(*B*), at this temperature is shown in Figure 2(c). A comparison of the simulated magnet and Nariki’s experimental results are shown in Figure 2(e) (f) (g), and (h), which show excellent agreement. Thus, the simulated bulk superconducting magnet represents realistic, state-of-the-art bulk materials.

In the following section, we use this modeling framework to explore the efficacy of the bulk superconductor-based MDT system, including the influence of lung-magnet distance, bulk superconductor properties, particle properties, and physiological or tumor structural parameters.

## 3. Results and discussion

The PDE, calculated on the surface of the cancer, is used to evaluate the guidance performance of the bulk superconductor-based MDT system. In this section, when simulating the influence of a single parameter change on MDT, the remaining parameters remain the same as the parameters in the model in the previous section. A single simulation takes about two hours to complete, which contains steady-state (magnetostatic) analysis for the magnetic field and flow field first, then transient analysis for particle tracing using the previous result. In this study, we utilized an AMD^®^ Ryzen 6900HX CPU @ 3.30 GHz, NVIDIA^®^ GeForce RTX 3070 Ti GPU, 16 GB RAM, Microsoft Windows 11 (64-bit) laptop. An exemplary animation of the simulation is provided in Supplementary Material 2.

### 3.1. Influence of particle properties on bulk superconductor-based MDT

During the MDT process, there may be differences in the size of different drug or drug carrier particles, which affects the drag and magnetic forces on the particles (see Equations (11) and (14)), thus changing the PDE value. Here, seven different particle diameters between 1–7 μm (Manshadi et al. 2019) are chosen to investigate the effect of particle size on PDE.

**Figure 3.**
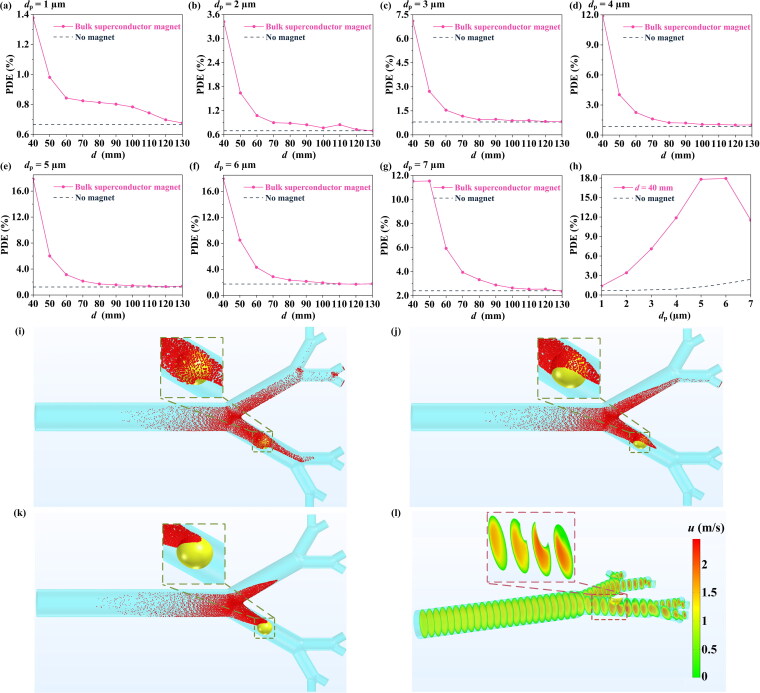
The effect of particle diameter, *d*_p_, on the PDE value, for various distances, *d*, between the bulk superconducting magnet and lung. (a) *d*_p_ = 1 µm, (b) *d*_p_ = 2 µm, (c) *d*_p_ = 3 µm, (d) *d*_p_ = 4 µm, (e) *d*_p_ = 5 µm, (f) *d*_p_ = 6 µm, (g) *d*_p_ = 7 µm. (h) Comparison of PDE values for different *d*_p_ values, for *d* = 40 mm, including the no magnet case. Simulated particle distributions for distance *d* = 40 mm for (i) *d*_p_ = 5 µm, (j) *d*_p_ = 6 µm, (k) *d*_p_ = 7 µm. (l) Air velocity field in the lung.

It can be seen from Figure 3(a)–(g) that, over a suitable range of *d*, for particles of different sizes the existence of the bulk superconducting magnet greatly improves the PDE value. For example, for a particle diameter of 6 µm, when *d* = 40 mm, the PDE can be increased to 17.94%, compared with 1.74% for no magnet. It should be noted here that without the action of the bulk superconducting magnet, the PDE gradually increases with increasing particle size, which is consistent with Manshadi’s results for a conventional permanent magnet (Manshadi et al. 2019). The main reason for this phenomenon is that as the particle diameter increases, the drag force gradually increases, increasing the tendency of particles to deflect toward the higher velocity region (the tumor surface region). In addition, when *d* = 40 mm, as the particle diameter increases, the PDE first increases and then decreases. This is because when the particle diameter ≤5 μm, with the increase in particle size, the magnetic force on the particles becomes stronger and more particles gather in the direction of the magnetic field, which greatly improves the PDE value. However, when the particle diameter ≥5 μm, the magnetic force is further strengthened, and the particles gather in advance of the tumor (Figure 3(i) (j) and (k)), causing the PDE value to decrease. Furthermore, as shown in Figures 3(j) and (k), the particles are mainly concentrated in the upper and upper left corner of the cancerous area, respectively. This is due to the fact that the higher air flow velocity at the inner side of the lung (see Figure 3(l)) causes the particles to deflect to the high velocity area under the action of the drag force (Manshadi et al. 2019).

Magnetic drugs or drug carriers may have differences in relative magnetic permeability depending on the particular material (Weidenfeller et al. 2008; Ni et al. 2021), thereby affecting the PDE value via Equation (14), the magnetophoretic force. Here, five different relative magnetic permeabilities are investigated over the range of 3–15 (Sulttan and Rohani 2023) to study the influence of particle relative magnetic permeability on PDE.

**Figure 4.**
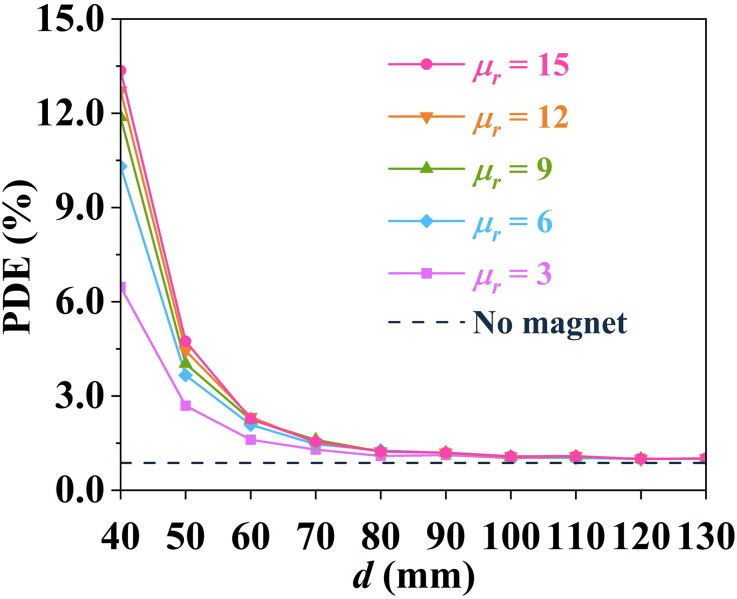
The effect of relative magnetic permeability on the PDE value, for various distances, *d.*

As shown in Figure 4, over a suitable range of *d*, particles with different relative magnetic permeabilities, the placement of the bulk superconductor magnet can partly increase the PDE value. For instance, the PDE could be as high as 13.36% while the relative magnetic permeability is 15, for *d* = 40 mm, whereas the no-magnet PDE is 0.87%. However, although the PDE value gradually increases with the increase in relative magnetic permeability of particles, the rate of increase in PDE drops since $\frac{\mu_{r,p}-\mu_{r}}{\mu_{r,p}+2\mu_{r}}$ in Equation (14) gradually approaches 1, causing the rate of increasing magnetic force to decrease. Besides, for the relative magnetic permeabilities investigated here, the PDE shows a decreasing trend as *d* increases, gradually approaching the same value as no magnet as the magnetophoretic force weakens.

### 3.2. Influence of physiological or tumor structural parameters on bulk superconductor-based MDT

In this section, we investigate the influence of physiological or tumor structural parameters on PDE, including the patient’s breathing rate and the tumor size, location and angle.

Firstly, the patient’s breathing rate can affect the drag force on the particles, thus influencing the PDE value via Equation (11). Based on Zhang et al. (2005) and Wang et al. (2024), four different breathing rates (*Q*) were considered to study the effect of breathing rate on bulk superconductor-based MDT.

**Figure 5.**
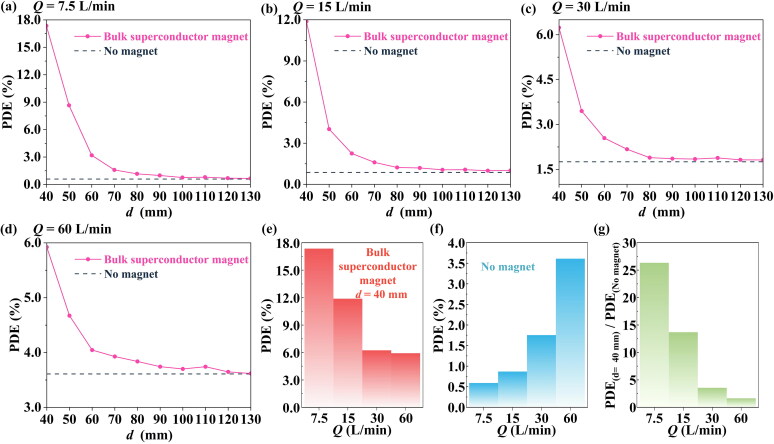
The effect of breathing rate on the PDE value, for various distances *d*. (a) *Q* = 7.5 L/min, (b) *Q* = 15 L/min, (c) *Q* = 30 L/min, (d) *Q* = 60 L/min. For different breathing rates, a comparison of (e) PDE at *d* = 40 mm, (f) PDE for no magnet, and (g) the ratio of PDE at *d* = 40 mm to PDE for no magnet.

It can be seen from Figure 5(a)–(d) that the existence of the bulk superconducting magnet can significantly improve the PDE value for different breathing rates over a suitable range of *d*. For example, when *Q* = 7.5 L/min and *d* = 40 mm, the PDE can be increased from 0.59% (no magnet) to 17.35%. In addition, Figure 5(e) and (f) show that when *Q* increases from 7.5 L/min to 60 L/min, the PDE value at *d* = 40 mm decreases, but also the PDE for no magnet actually increases. This makes the ratio between the two PDE values (with and without the bulk superconducting magnet) at *d* = 40 mm decrease sharply (see Figure 5(g)). This is because, without the magnetic field effect, the main reason for particle deposition in the cancer area is inertial impingement (Inthavong et al. 2010; Liu et al. 2023). The increase in breathing rate greatly improves the inertial forces, which enhances the inertial impingement and subsequently increases the PDE. On the other hand, the particles also need a larger magnetic force to alter their motion when being subjected to a greater inertial force.

Next, in clinical cases, the size of tumor tissue varies significantly among patients, so five different *r*/*R*_G1_ values (Manshadi et al. 2019; Sabz et al. 2019) were considered to investigate the effect of cancer tissue size on PDE.

**Figure 6.**
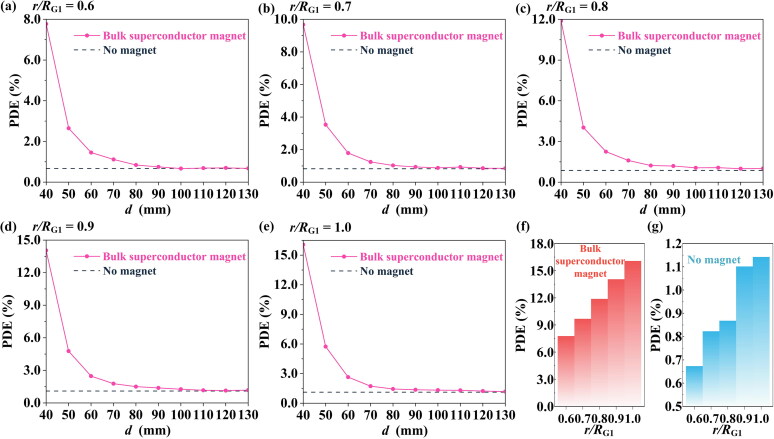
The effect of tumor size on the PDE value, for various distances *d*. (a) *r*/*R*_G1_ = 0.6, (b) *r*/*R*_G1_ = 0.7, (c) *r*/*R*_G1_ = 0.8, (d) *r*/*R*_G1_ = 0.9, (e) *r*/*R*_G1_ = 1. For different tumor sizes, a comparison of PDE values at *d* = 40 mm (f) with and (g) without the bulk superconducting magnet.

Figure 6(a)–(e) show that, under the effect of the bulk superconducting magnet, the PDE value could be significantly improved over a suitable range of *d* for different sizes of tumor. For example, when *r*/*R*_G1_ = 1 and *d* = 40 mm, the PDE is 16.06%, which is much higher than that for no magnet. As shown in Figure 6(f) and (g), since increasing the surface area of the cancerous region increases the probability of contact between the particles and the tumor surface, with increasing *r*/*R*_G1_, the PDE at *d* = 40 mm and in the no magnet case have both increased.

**Figure 7.**
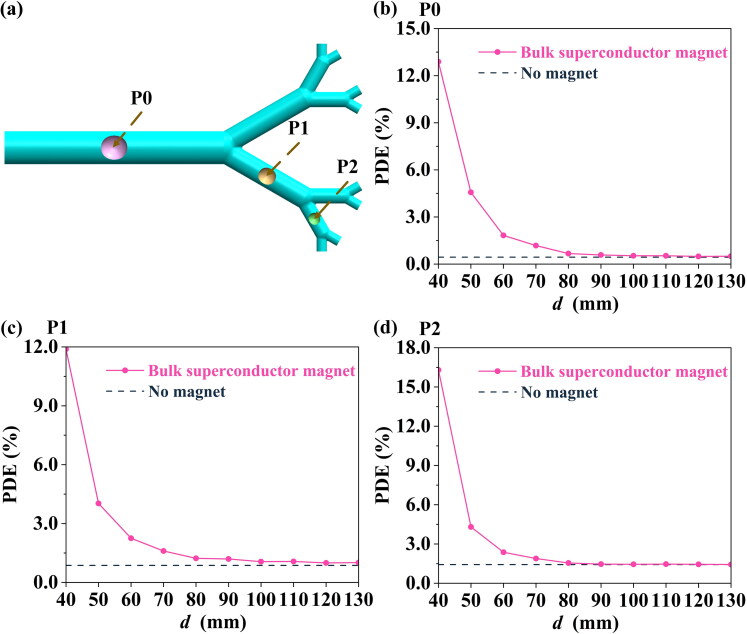
(a) Schematic diagram of the different tumor locations investigated. The effect of tumor location on the PDE value: (b) P0, (c) P1, and (d) P2, for various distances, *d.*

For different locations of tumor (P0, P1, and P2; see Figure 7(a)), the placement of the bulk superconducting magnet can dramatically improve the PDE over a suitable range of *d*. For instance, for *d* = 40 mm, an increase in the PDE value from 0.44% (no magnet) to 12.89% at location P0 (see Figure 7(b)); an increase in the PDE value from 0.86% (no magnet) to 11.88% at P1 (see Figure 7(c)); and an increase in the PDE value from 1.42% (no magnet) to 16.30% at P2 (see Figure 7(d)).

**Figure 8.**
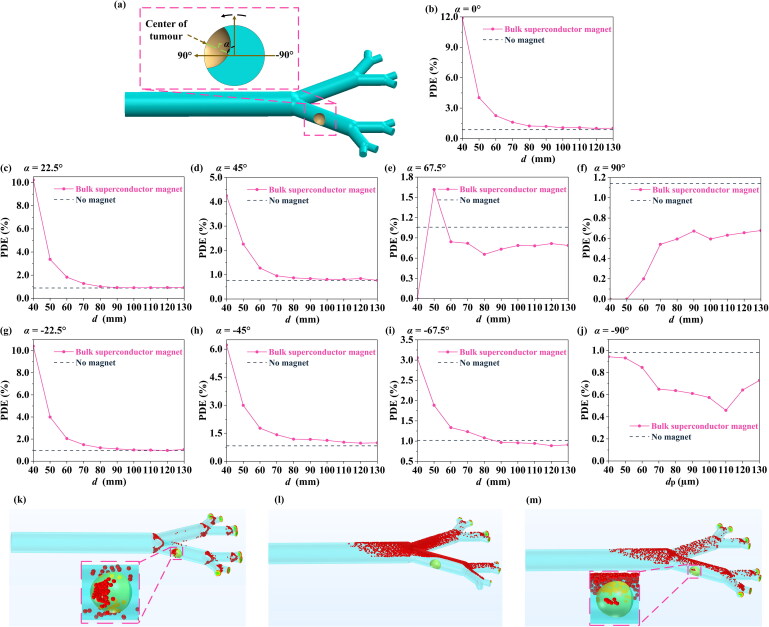
(a) Schematic diagram defining the tumor angle, *α*. The effect of tumor angle on the PDE value, for various distances *d*. (b) *α* = 0°, (c) *α* = 22.5°, (d) *α* = 45°, (e) *α* = 67.5°, (f) *α* = 90°, (g) *α* = −22.5°, (h) *α* = −45°, (i) *α* = −67.5°, (j) *α* = −90°. Simulated particle distributions for (k) no magnet, (l) *d* = 40 mm, and (m) *d* = 60 mm, while *α* = 90°.

So far, in this paper, we have focused on the tumor tissue growing on the upper surface of the lung (or conversely, the bottom surface of the lung, for which the bulk superconducting magnet could be located at the back of the body), so placement of the bulk superconducting magnet can coincide better with the tumor tissue and capturing the magnetic particles. However, in practice, a tumor tissue may be generated anywhere in the lung (Satpathy et al. 2021; Restrepo et al. 2023). In consideration of this, to investigate the generality of the MDT process using the bulk superconducting magnet, we define the tumor angle *α* (as shown in Figure 8(a), deflection angle between the center of the tumor and the initial position) and calculate nine sets of PDE values for different angles.

As shown in Figure 8(c), 8(d) and 8(g)–(i), while −67.5° ≤ *α* ≤ 45°, the presence of the bulk superconducting magnet can improve the PDE value over a reasonable range of *d*. However, Figure 8(e), 8(f) and 8(j) show that, when α = −90°, 67.5° or 90°, the existence of the bulk superconducting magnet could lead to a decrease in PDE, which is even less effective than the no magnet case. In this case, the bulk superconducting magnet ends up deflecting particles that would originally be able to stick to the tumor surface in the direction of the magnetic field, thereby failing to stick to the tumor surface and reducing PDE (Figure 8(k)–(m)). In this case, the PDE value can be improved by adjusting the position of the bulk superconducting magnet (see Supplementary Material 1.3).

### 3.3. Influence of bulk superconducting magnet properties on MDT

The dimensions of the bulk superconducting magnet have a significant impact on the magnetic field strength and distribution, as described by Equations (3) and (4), which will in turn affect the PDE of the MDT process. In practical fabrication of bulk superconductors (and with reference to Equation (4)), their dimensions usually maintain a certain aspect ratio (Eisterer et al. 2006; Shi et al. 2019). Therefore, in this section, bulk superconducting magnet models with the various dimensions detailed in Table 3 were built to study the relationship between bulk superconductor size and PDE.

**Table 3.** Dimensions of the bulk superconductor magnet investigated and the simulated magnetic field strength for each one.

Radius, *a* (mm)	Thickness, *t* (mm)	Peak surface magnetic field, *B*_T_ (T)
15	9	2.11
25	15	3.02
32.5	19	3.42
40	23	3.70
50	29	3.99

**Figure 9.**
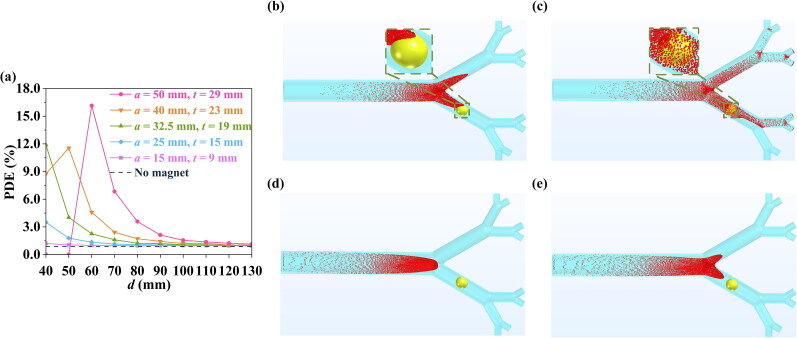
(a) The effect of distance *d* and bulk superconducting magnet dimensions on the PDE value. Simulated particle distributions for bulk dimensions *a* = 40 mm, *t* = 23 mm for (b) *d* = 40 mm, (c) *d* = 50 mm; and bulk dimensions *a* = 50 mm, *t* = 29 mm for (d) *d* = 40 mm, (e) *d* = 50 mm.

As shown in Figure 9(a), the presence of the bulk superconducting magnet usually improves the PDE of particles for the different superconducting magnet sizes over a suitable range of *d*. For instance, when the magnet size parameters are set to *a* = 50 mm, *t* = 29 mm, and *d* = 60 mm, the PDE can be increased to 16.15%, compared to 0.87% for no magnet. For *a* = 50 mm, *t* = 29 mm and *a* = 40 mm, *t* = 23 mm, two sizes of bulk superconducting magnet, as *d* increases, the PDE first increases and then decreases (see Figure 9(a)). This is because when the size of the magnet is too large, the magnetic field strength and distribution are greatly enhanced. The particles in fact gather before reaching the cancerous area due to the magnetic force (see Figure 9(b)–(e)), which causes the PDE to decrease to as low as zero when the stronger bulk superconducting magnet is closer to the cancerous area. For this phenomenon, the magnet position can then be changed to increase the PDE (see Supplementary Material 1.4).

For bulk superconducting magnets, changes in material composition and processing conditions, as well as the cryogenic operating temperature, affect the *J*_c_(B) property (Nariki et al. 2005; Ainslie et al. 2016; Antončík et al. 2024), which then changes the magnetic field available from the bulk superconducting magnet after magnetization (see Equation (3)). Therefore, the PDE value will change. Here, three *J*_c_(B) properties for representative Gd-Ba-Cu-O bulk samples at different operating temperatures were chosen for further simulations. The details of the material composition and simulated magnetic fields are shown in Table 4. Each of the *J*_c_(B) curves are provided in Figure S4 in Supplementary Material 1.5.

**Table 4.** Representative bulk superconducting magnets at different operating temperatures and their simulated magnetic field strengths.

No	Materials	Operating temperature (K)	*B_T_* (T)
M1 (Nariki et al. 2005)	Gd-Ba-Cu-O with 0.5 wt% Pt and 20 wt% Ag_2_O	77	3.42
M2 (Ainslie et al. 2016)	Gd-Ba-Cu-O with 15 wt% Ag_2_O	60	6.95
		50	10.80

**Figure 10.**
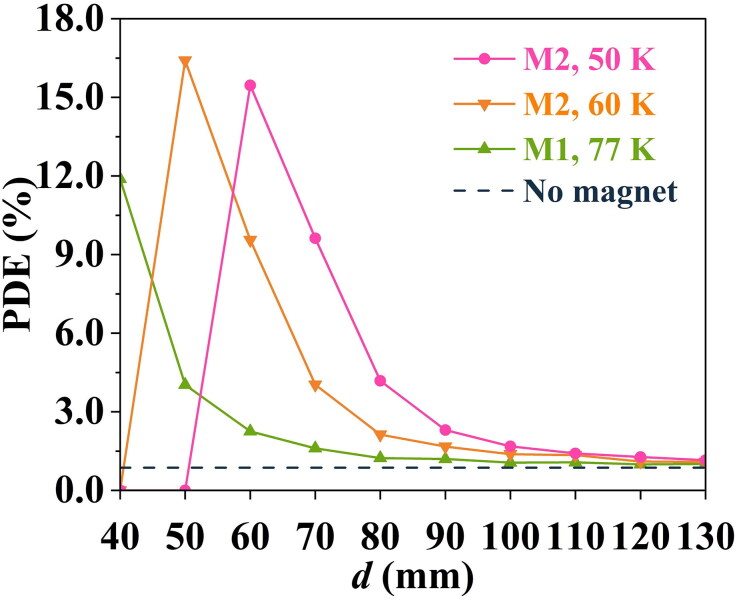
The effect of the cryogenic operating temperature change on the PDE value, for various distances.

In this case, as shown in Figure 10, the bulk superconducting magnet usually improves the PDE value at different cryogenic operating temperatures over a suitable range of *d*. For example, the PDE could be improved to 16.41% at 50 K, which is much higher than the no magnet value of 0.87%. Similarly, for an operating temperature of 50 or 60 K, the PDE first increases, then drops with increasing *d*. It is clear that increasing the magnetic field strength with an increased *J*_c_ due to the lower operating temperature can increase the PDE value, but this can also cause particles to gather in advance of the cancerous area by the magnetic force, causing the PDE value to decrease even to zero.

## 4. Conclusion

In this work, magnetic drug targeting (MDT) using a bulk superconducting magnet has been explored for the treatment of primary bronchial cancer. A three-dimensional numerical simulation model was built in COMSOL Multiphysics, based on the Weibel model of the lung, interacting with a magnetized bulk superconductor. The model was used to study the influence of lung-magnet distance, bulk superconductor properties, particle properties, and physiological or tumor structural parameters on capture efficiency. The results show that the use of the bulk superconducting magnet can effectively improve the capture efficiency of magnetic drugs or drug carriers within a suitable distance (especially 40–80 mm away from the lesion area). A particle deposition efficiency (PDE) value of over 10% can be readily achieved when *d* = 40 mm, whereas the no-magnet PDE is usually less than 1–2%. The results prove that bulk superconducting magnets have great potential to realize the guidance of magnetic drugs or drug carriers from outside the body, which could achieve safer noninvasive magnetic-targeted medical treatment for lung cancer. In addition, the distribution and deposition patterns of particles are discussed for the various cases investigated. The results also illustrate that when the bulk superconducting magnet is directly opposite the tumor, although the magnetic field strength can be modified by changing the magnet properties (e.g. dimensions or cryogenic operating temperature), the PDE does not always increase due to the fact that a stronger magnet force could actually gather particles before the cancerous area. However, a stronger magnetic field increases the effective distance *d*.

In summary, this numerical simulation framework provides a basis for more detailed design and optimization of MDT using a bulk superconducting magnet, which lays the foundation for further biological experiments and future clinical translation for lung cancer MDT treatment. However, this research is limited by the simplified lung and cancer models, which can be more complicated in reality and to some degree patient specific. Meanwhile, the relationship between the 3D spatial position of the bulk superconducting magnet and deposition efficiency needs to be explored further to improve such an MDT system. We believe that, by using imaging and importing CAD models as input data and intelligent algorithms to seek the suitable spatial positions for the bulk superconducting magnet, it could provide an opportunity for high-efficiency MDT tailored to a specific lung cancer patient in the future.

## Supplementary Material

Supplemental Material

## Data Availability

The data that support the findings of this study are available from the corresponding author, upon reasonable request.
