# Supplementary material for: Numerical simulation of magnetic drug targeting for lung cancer therapy using a bulk superconducting magnet
Source: Drug Deliv. 2025 Apr 29;32(1):2490836. doi: 10.1080/10717544.2025.2490836 (PMC12042242; doi:10.1080/10717544.2025.2490836)
Supplement: Supplemental Material [file IDRD_A_2490836_SM1586.zip › Supplementary Material 2/Supplementary material 1 280225.docx]

**Supplementary material 1.1**

**Table S1**

Research on conventional magnets for magnetic drug targeting.

| Category | | Reference | Max. magnetic field strength | Experiment |
| --- | --- | --- | --- | --- |
| Conventional  permanent magnets | Nd magnet | Song et al. [1] | Not given | Simple in vitro model |
|  | Square and ring shape NdFeB magnet | Depalo et al. [2] | 1.17 T | Simple in vitro model |
|  | Ferrite and Nd magnet | Bernad et al. [3] | Ferrite: 0.45 T  Nd: 1.47 T | Simple in vitro model and 2D computer simulation |
|  | NdFeB magnet | Ebrahimi et al. [4] | 0.3 T, 0.5 T | 2D computer simulation |
|  | NdFeB magnet | Lunnoo et al. [5] | 1.4 T | 2D computer simulation |
|  | Cylindrical-shaped permanent magnet | Varmazyar et al. [6] | 0.6 T | 2D computer simulation |
|  | NdFeB magnet | Sharma et al. [7] | 0.4 T (2.5 cm) | 2D computer simulation |
|  | Permanent magnet | Manshadi et al. [8] | About 2 T (However, it does not match the actual magnetic field strength of permanent magnets) | 2D computer simulation |
|  | Several permanent magnets | Aryan et al. [9] | 0.4 T for single magnet | 3D computer simulation |
|  | Annular array of permanent magnets | Babincova et al. [10] | About 2 T | 2D computer simulation |
|  | Halbach magnet, two different sizes of cylindrical permanent magnets | Shen et al. [11] | Not given | Biological experiments (in vivo) |
|  | Multiple layer Halbach magnet | Barnsley et al. [12] | About 1.3 T | Simple in vitro model |
|  | Bar type Halbach magnet | Ranjbari et al. [13] | About 1.6 T | 3D computer simulation |
| Static field electromagnet | Current-carrying wires | Sodagar et al. [14] | About 1.7 T | 3D computer simulation |
|  | Current-carrying wires | Larimi et al. [15] | About 1.8 T | 2D computer simulation |
|  | U-shaped electromagnet | Pondman et al. [16] | About 0.08 T | Biological experiments (in vitro and vivo) |
|  | Electromagnet | Hoke et al. [17] | 1.431 T | 3D computer simulation |
|  | Open pot type electromagnet | Alexiou et al. [18] | 2.2 T | Computer simulation |
|  | Tip-top electromagnet | Voronin et al. [19] | 0.62 T | Biological experiments (in vitro and vivo) |

**Supplementary material 1.2**

This section will explain the selection principles for the range of *d*. Assuming that the bronchus is located in the center of the chest and the chest depth is set to *l_1_* (see Fig. S1), then in a static state, the distance between the skin surface and the bronchus would be *l_1_*/2. In actual clinical procedures, a certain space can be pressed down on the chest [20, 21]. As a result, this could decrease the *d* to less than *l_1_*/2 while using the bulk superconducting magnet to attract the drug. Based on the data in Table S2, the integer *d* was selected from 40 mm to 130 mm.


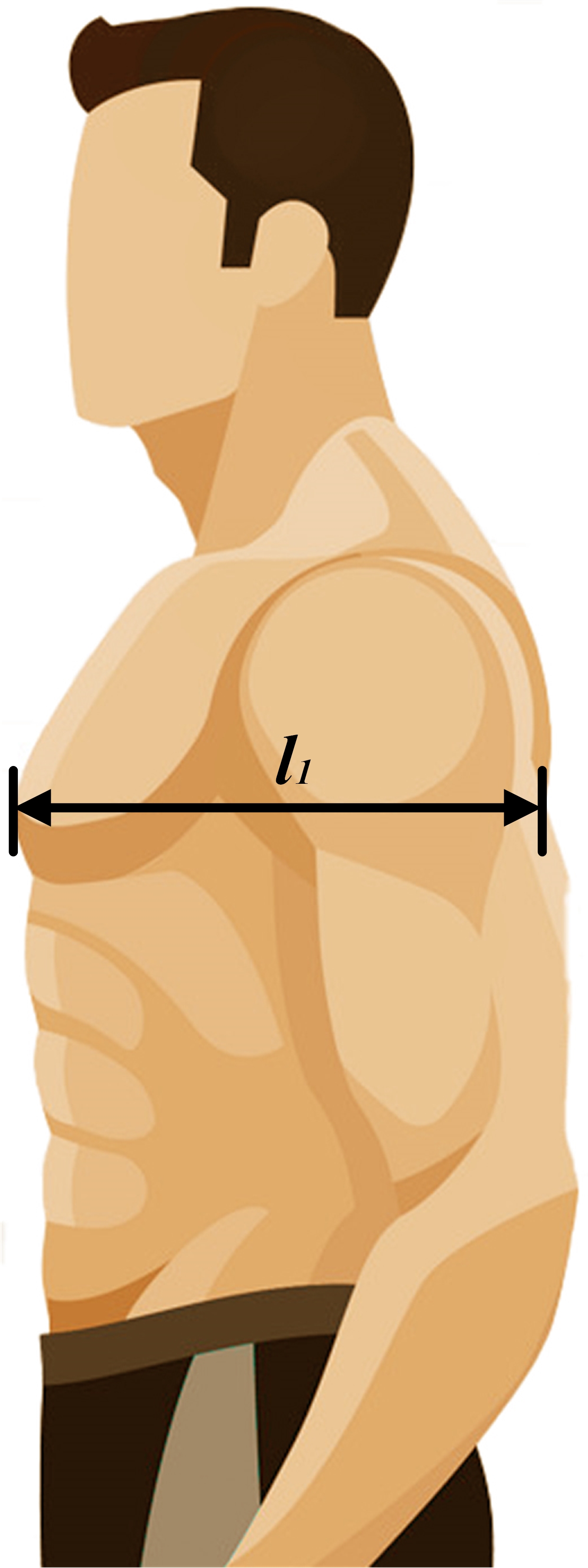


**Fig. S1.** Schematic diagram of a human upper body.

**Table S2**

Chest depth (*l_1_*) data of different age groups and genders in China and Japan.

| Age group | Gender | Nationality | *l_1_* | *l_1_*/2 | Average value | Reference |
| --- | --- | --- | --- | --- | --- | --- |
| 4-6 | Male | Chinese | 126-184 | 64-92 | - | [22] |
|  | Female |  | 121-175 | 61-87.5 | - |  |
| 11-12 | Male |  | 146-249 | 73-124.5 | - |  |
|  | Female |  | 141-234 | 70.5-117 | - |  |
| 16-17 | Male |  | 174-272 | 87-136 | - |  |
|  | Female |  | 165-240 | 82.5-120 | - |  |
| 18-70 | Male |  | 172-270 | 86-135 | - | [23] |
|  | Female |  | 168-265 | 84-132.5 | - |  |
| 20-30 | Male | Japanese | 173-261 | 86.5-130.5 | 209.7 | [24, 25] |
| 21-35 | Female |  | 193-283.5 | 96.5-141.75 | 220.7 |  |
|  |  |  | 154.5-255(Underbust) | 77.25-127.5 | 184.9 |  |

**Supplementary material 1.3**

This section shows that changing the position of the bulk superconducting magnet (see Fig. S2(a)) can change the PDE (when *α* = 90° and *d* = 40 mm). The magnetic particles’ (drugs or drug carriers) distribution for two different positions (X = 25 mm, Y = 10 mm and X = 40 mm, Y = 10 mm) are shown in Figs. S2(b) and S2(c), respectively. The corresponding PDE values are provided in Table S3.





**Fig. S2.** Schematic diagram of the axis for the bulk superconducting magnet moving. Schematic diagram of simulation results for (b) X = 25 mm, Y = 10 mm and (c) X = 40 mm. Y = 10 mm, when *α* = 90°, and *d* = 40 mm.

**Table S3**

PDE values for the bulk superconducting magnet centered at different positions (X, Y; see Fig. S2(a)), when *a* = 50 mm, *t* = 29 mm, and *d* = 40 mm.

| X (mm) | Y (mm) | PDE (%) |
| --- | --- | --- |
| 0 | 0 | 0 |
| 25 | 10 | 2.39 |
| 40 | 10 | 1.62 |

**Supplementary material 1.4**

This sections also shows that changing the position of the bulk superconducting magnet can change the PDE (when *a* = 50 mm, *t* = 29 mm, and *d* = 40 mm). The magnetic particles’ (drugs or drug carriers) distribution for two different positions X = 27 mm, Y = 0 mm is shown in Fig. S3. The corresponding PDE value is provided in Table S4.


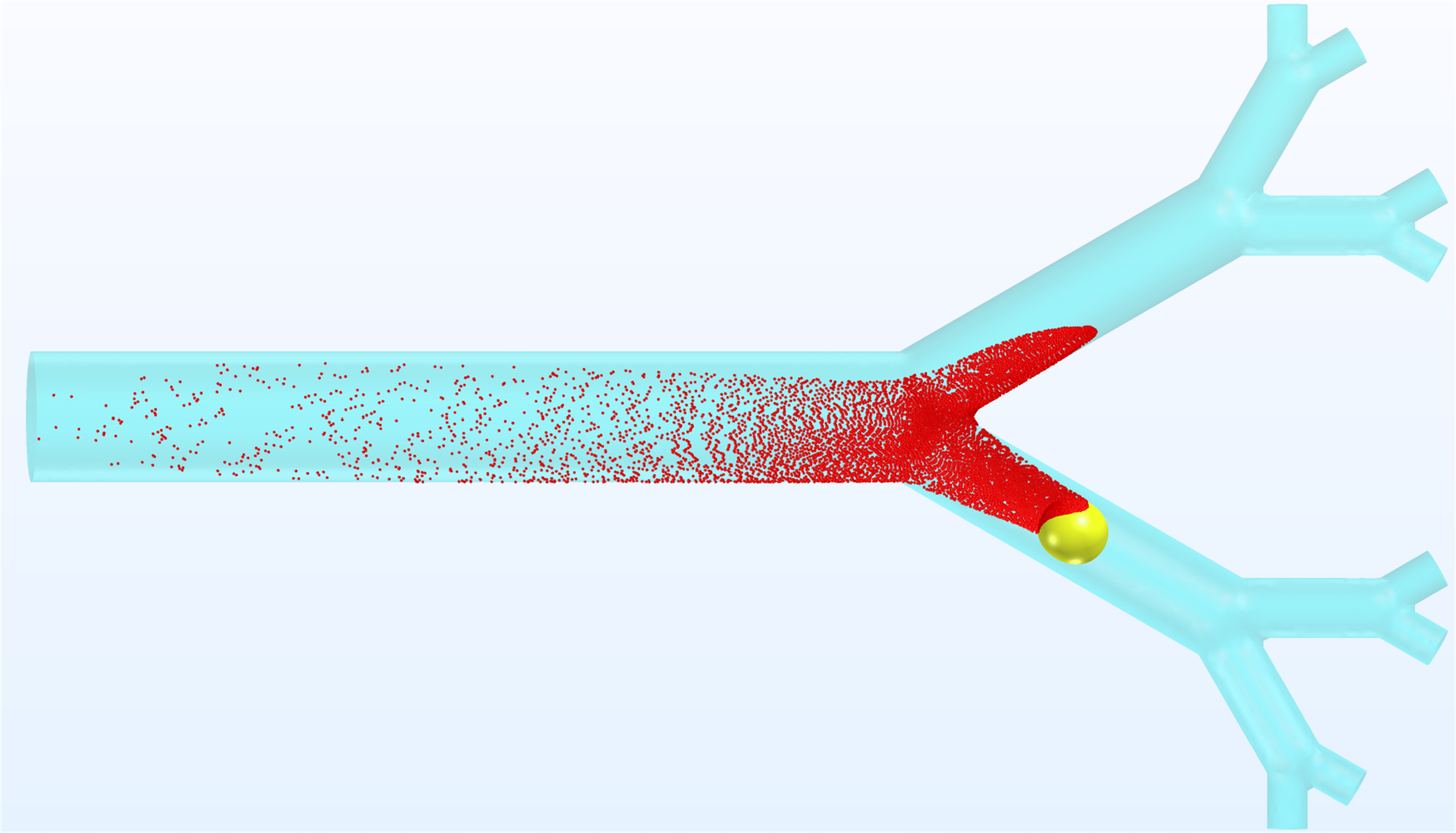


**Fig. S3.** Schematic diagram of simulation results for X = 27 mm, Y = 0 mm, when *a* = 50 mm, *t* = 29 mm, and *d* = 40 mm.

**Table S4**

PDE value for the bulk superconducting magnet centered at X = 27 mm. Y = 0 mm, when *a* = 50 mm, *t* = 29 mm, and *d* = 40 mm.

| X (mm) | Y (mm) | PDE (%) |
| --- | --- | --- |
| 27 | 0 | 16.55 |

**Supplementary material 1.5**


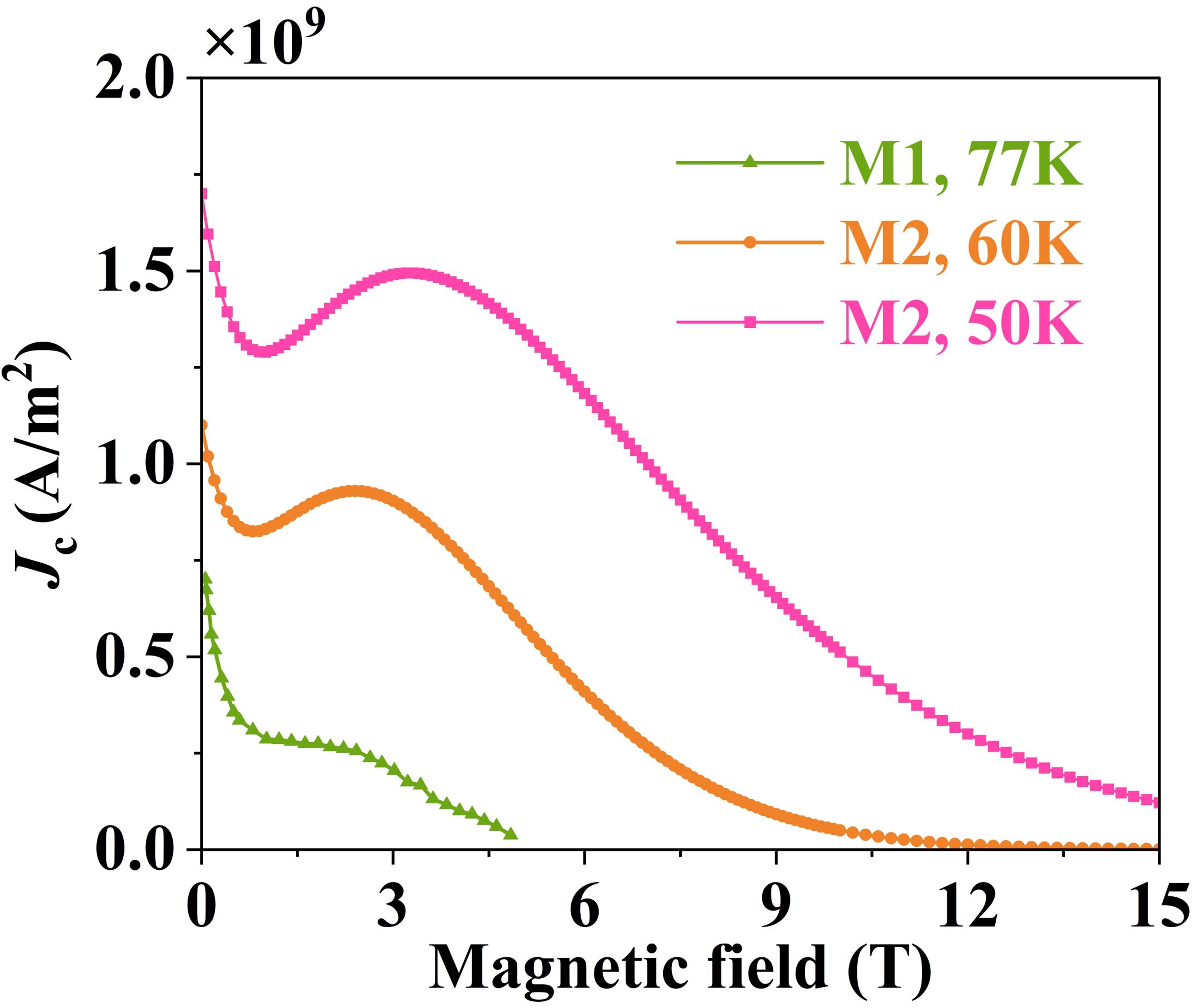


**Fig. S4.** *J*_c_(*B*) curves of different materials at different operating temperatures [26, 27].

**Supplementary material references**

[1] Song W, Su X, Gregory D A, et al. Magnetic alginate/chitosan nanoparticles for targeted delivery of curcumin into human breast cancer cells. Nanomaterials. 2018, 8(11): 907.

[2] Depalo N, Iacobazzi R M, Valente G, et al. Sorafenib delivery nanoplatform based on superparamagnetic iron oxide nanoparticles magnetically targets hepatocellular carcinoma. Nano Res. 2017, 10: 2431-2448.

[3] Bernad S I, Bernad E. Magnetic Forces by Permanent Magnets to Manipulate Magnetoresponsive Particles in Drug-Targeting Applications. Micromachines, 2022, 13(11): 1818.

[4] Ebrahimi S, Shamloo A, Alishiri M, et al. Targeted pulmonary drug delivery in coronavirus disease (COVID-19) therapy: A patient-specific in silico study based on magnetic nanoparticles-coated microcarriers adhesion. Int. J. Pharm. 2021, 609: 121133.

[5] Lunnoo T, Puangmali T. Capture efficiency of biocompatible magnetic nanoparticles in arterial Flow: a computer simulation for magnetic drug targeting. Nanoscale Res. Lett. 2015: 10: 426.

[6] Varmazyar M, Habibi M R, Amini M, et al. Numerical simulation of magnetic nanoparticle-based drug delivery in presence of atherosclerotic plaques and under the effects of magnetic field. Powder Technol, 2020, 366: 164-174.

[7] Sharma S, Katiyar V K, Singh U. Mathematical modelling for trajectories of magnetic nanoparticles in a blood vessel under magnetic field. J. Magn. Magn. Mater, 2015, 379: 102-107.

[8] Manshadi M K D, Saadat M, Mohammadi M, et al. Delivery of magnetic micro/nanoparticles and magnetic-based drug/cargo into arterial flow for targeted therapy. Drug Deliv, 2018, 25(1): 1963-1973.

[9] Aryan H, Beigzadeh B, Siavashi M. Euler-Lagrange numerical simulation of improved magnetic drug delivery in a three-dimensional CT-based carotid artery bifurcation. Comput Methods Programs Biomed. 2022, 219: 106778.

[10] Babincova M, Babinec P. Magnetic drug delivery and targeting: principles and applications. Biomed Pap Med Fac Univ Palacky Olomouc Czech Repub. 2009, 153(4): 243-50.

[11] Shen W B, Anastasiadis P, Nguyen B, et al. Magnetic enhancement of stem cell–targeted delivery into the brain following MR-guided focused ultrasound for opening the blood–brain barrier. Cell Transplant. 2017, 26(7): 1235-1246.

[12] Barnsley L C, Carugo D, Owen J, et al. Halbach arrays consisting of cubic elements optimised for high field gradients in magnetic drug targeting applications. Phys. Med. Biol. 2015, 60(21): 8303.

[13] Ranjbari L, Zarei K, Alizadeh A, et al. Three-dimensional investigation of capturing particle considering particle-RBCs interaction under the magnetic field produced by an Halbach array. J Drug Deliv Sci Technol. 2023, 79: 104046.

[14] Sodagar H, Shakiba A, Niazmand H. Numerical investigation of drug delivery by using magnetic field in a 90-degree bent vessel: a 3D simulation. Biomech Model Mechanobiol. 2020, 19: 2255-2269.

[15] Larimi M M, Ramiar A, Ranjbar A A. Numerical simulation of magnetic nanoparticles targeting in a bifurcation vessel. J. Magn. Magn. Mater. 2014, 362: 58-71.

[16] Pondman K M, Bunt N D, Maijenburg A W, et al. Magnetic drug delivery with FePd nanowires. J. Magn. Magn. Mater. 2015, 380: 299-306.

[17] Hoke I, Dahmani C, Weyh T. Design of a high field gradient electromagnet for magnetic drug delivery to a mouse brain. Proceedings of the COMSOL Conference. 2008.

[18] Alexiou C, Diehl D, Henninger P, et al. A high field gradient magnet for magnetic drug targeting. IEEE Trans. Appl. Supercond 2006, 16(2): 1527-1530.

[19] Voronin D V, Sindeeva O A, Kurochkin M A, et al. In vitro and in vivo visualization and trapping of fluorescent magnetic microcapsules in a bloodstream. ACS Appl. Mater. Interfaces. 2017, 9(8): 6885-6893.

[20] Agostinucci J M, Weisslinger L, Marzouk N, et al. Relation between chest compression rate and depth: the ENFONCE Study. Eur J Emerg Med, 2021, 28(5): 352-354.

[21] Beesems S G, Hardig B M, Nilsson A, et al. Force and depth of mechanical chest compressions and their relation to chest height and gender in an out-of-hospital setting. Resuscitation, 2015, 91: 67-72.

[22] Human dimensions of Chinese minors. <https://openstd.samr.gov.cn/bzgk/gb/newGbInfo?hcno=6E21BA94A863EE453622638A6C98DDAF>, 2024 (accessed 3 June 2024).

[23] Human dimensions of Chinese adults. <https://openstd.samr.gov.cn/bzgk/gb/newGbInfo?hcno=B19DCCA575D9406856ABF87A511EE11F>, 2024 (accessed 3 June 2024).

[24] AIST Human Body Dimensions Database 1991-92. <https://www.airc.aist.go.jp/dhrt/91-92/index.html> , 2024 (accessed 3 June 2024).

[25] AIST/HQL Human Body Dimensions and Shape Database 2003. <https://www.airc.aist.go.jp/dhrt/fbodydb/index.html>, 2024 (accessed 3 June 2024).

[26] Nariki S, Sakai N, Murakami M, Melt-processed Gd–Ba–Cu–O superconductor with trapped field of 3 T at 77 K*.* Superconductor Science and Technology, 2004. 18(2): S126.

[27] Ainslie M, et al., Enhanced trapped field performance of bulk high-temperature superconductors using split coil, pulsed field magnetization with an iron yoke*.* Superconductor Science and Technology, 2016. 29(7): 074003.
